# The In-Out dispositional affective style questionnaire (IN-OUT DASQ): an exploratory factorial analysis

**DOI:** 10.3389/fpsyg.2014.01005

**Published:** 2014-09-11

**Authors:** Viridiana Mazzola, Giuseppe Marano, Elia M. Biganzoli, Patrizia Boracchi, Tiziana Lanciano, Giampiero Arciero, Guido Bondolfi

**Affiliations:** ^1^Swiss Center for Affective Sciences – University of GenevaGeneva, Switzerland; ^2^Department of Clinical Sciences and Community Healt, University of MilanMilan, Italy; ^3^Unit of Medical Statistics and Biometry, Fondazione Istituto Di Ricovero e Cura a Carattere Scientifico Istituto Nazionale dei TumoriMilan, Italy; ^4^Institute of Post-Rationalist PsychologyRome, Italy; ^5^Department of Education, Psychology, Communication Science, University of BariBari, Italy; ^6^Department of Psychiatry, Hôpitaux Universitaires de GenèveGeneva, Switzerland

**Keywords:** affective dispositions, inwardness, outwardness, social behavior, social affective neuroscience

## Abstract

The issue of individual differences has always been an important area of research in psychology and, more recently, neuroimaging. A major source of interindividual variability stems from differences in basic affective dispositions. In order to make a contribution to this field of research, we have developed a new type of assessment – the In-Out dispositional affective style questionnaire (IN-OUT DASQ) – to measure the proneness between two different ways of feeling situated: a predominantly body-bound one in the case of the inward tendency and an externally anchored one in the case of the outward tendency (Arciero and Bondolfi, 2009). The IN-OUT DASQ contains two scales of seven items each, Self-centric engagement (SCE) and Other-centric engagement (OCE), as a disposition index for inwardness and outwardness respectively. The exploratory factor analysis in sample 1 (*n* = 292) confirmed a two-factor solution. Confirmatory factor analysis in sample 2 (*n* = 300) showed the good fit of this two-factor model. Next, we examined construct validity also investigating the correlations between the IN-OUT DASQ, the Big Five Questionnaire and the Positive and Negative Affect Schedule in sample 3 (*n* = 153). The SCE and OCE scales had robust internal consistency and reliability, though the capacity to discriminate higher inward and outward participants was stronger in SCE. Although further validation research is required, the present study suggests the IN-OUT DASQ has the potential to be a measurement tool for detecting individual differences in social behavior and social affective neuroscience.

## INTRODUCTION

Personality and social psychology have taken up conceptual and methodological challenges to understand how individual differences affect social behaviors (Leary and Hoyle, 2009). On one hand, there is an ongoing debate about the conceptualization of the Self – with respect to the notions of continuity, unity, and privacy – as a complex system (Cloninger et al., 1993; Thelen and Smith, 1994; Cloninger and Svrakic, 1999; Thelen, 2002; Lewis, 2005), vs. the self as a social construction (Gergen, 1991, 1999; Lifton, 1993). On the other hand, the long standing debate on affect and cognition primacy (Lazarus, 1982; Zajonc, 1984) still raises questions on emotional experience and its affective, cognitive, and bodily components (Griffiths, 1997; Damasio, 1999; Prinz, 2004; Barrett, 2013; Scherer, 2013; Levenson, 2014).

In recent years, this subject has been expanded to include neuroimaging techniques. Indeed, it has become possible to identify the relationship between neural mechanisms underlying behaviors and some individual characteristics (Hariri, 2009), showing how individual differences are rooted in brain anatomy and functional connectivity (Mueller et al., 2013). On the other hand, a great source of interindividual variability may be due also to differences in general affective dispositions (Bertolino et al., 2005; Rubino et al., 2007; Mazzola et al., 2010). Here we propose a new means of assessing two dispositional affective styles, inward and outward, in order to contribute to the study of individual differences both in affective neuroscience and social psychology.

As Heidegger (1962, §29 p. 176) stated, a mood assails us. It comes neither from outside nor from inside, but arises out of Being-in-the-World, as a way of such Being. According to Heidegger, an emotional state is not in the head, but is rather the elementary manifestation of our being situated: of our being immersed and absorbed in our comportment toward the concerns of our everyday lives and activities (Arciero, 1989; Arciero et al., 2004; Arciero and Bondolfi, 2009). Therefore, feeling oneself in a certain emotional situation ties the way I perceive myself as living to what addresses me in that situation. Several authors consider the dispositional affect to be the predominant modality of emotional engagement with the self and with the environment (Merleau-Ponty, 1962; Gallagher, 2007a,b; Gallagher and Hutto, 2008). As Zajonc (1984) stated, the individual is never without being in some emotional state.

In the present study, the concept of dispositional affect emphasizes the need to account for the way in which different persons, in dealing with others and the different circumstances of everyday life, feels situated in the environment (Arciero, 1989, 2002; Mahoney et al., 1995). Being emotionally situated thus corresponds to a tension which is born and which is continually renewed in the sphere of social and practical engagement but which, at the same time, reflects and actualizes the story of the emoter (Arciero and Guidano, 2000; Arciero, 2006). This encounter enables the emoter to orient himself in the world each and every time, grasping those elements of significativity and the geography of saliences, generating the possibilities of action which are most attuned to the emerging contexts (Gallagher, 2007a,b; Gallagher and Hutto, 2008). Therefore, situational engagement re-sets, each and every time, the vital space in terms of the possible actions that circumstances require (Mazzola et al., 2013). Viewed in this light, already at a pre-reflective level, e-moting is the embodied meaning of the ongoing situation. According to this perspective, emotions take shape from one’s engagement with the other in ongoing situations (Arciero and Bondolfi, 2009).

The nature of emotions has remained one of the longest standing debates in the biological and social sciences. Indeed, there are several perspectives on basic and non-basic emotions. There is a general agreement that basic emotions must have direct causal powers over motivation and behavior, at least in early developmental stages. In all cases, this argument is based on evolutionary principles (Tracy and Randles, 2011). If an emotion evolved to facilitate adaptive coping with specific ecological challenges, then that emotion would need to cause and motivate appropriate behavioral and physiological responses to address the relevant challenges (Ekman and Cordaro, 2011). However, as individuals develop higher level cognitive and social capacities that allow for emotion regulation, these causal effects become probabilistic, merely increasing the likelihood of emotion-congruent behavior. On the other hand, things seem to move in a different direction if the recurrent stimuli the emoter is exposed to in his interactions with significant people do not elicit specific responses. This type of reciprocity is based on a “mediated” affective engagement and gives rise to a predictability which must perforce be anchored in the external source of stimulation.

This way of emotioning may be described by using the words employed by Draghi-Lorenz et al. (2001; p. 295) to describe non-basic emotions: “They seem to owe their distinct status to being de facto always and necessarily socially aware emotions.” While this produces a recognition of one’s own emotional experiences stemming from an initial focalization on the other, it hinders focusing attention on one’s own internal states. It is as if evolution had not only prepared the organism to produce automatic appraisal of stimuli, which are relevant to the maintenance of adaptation, but rather also to a new set of emotions arose to deal with critical social needs (Tracy and Randles, 2011).

One could sum this up by suggesting that by the recurrent affective engagement with significant people the emoter comes to acquire over time a better knowledge of families of basic emotions rather than non-basic, or vice versa. In accordance with these assumptions, we outlined the two general affective dispositions, inward and outward. The distinction between inward and outward tendencies was seen to be based on whether basic or non-basic emotions are prevalently elicited, as well as on the combination between the two dimensions they define (Arciero et al., 2004; Arciero, 2006). The two dimensions express different ways of feeling situated: a predominantly body-bound one in the case of the inward tendency, an externally anchored one in the case of the outward tendency (Arciero, 1989; Arciero and Bondolfi, 2009; Mazzola et al., 2010). Within the framework of these tendencies each individual can then be seen to possess a specific emotional makeup. This makeup may vary across different periods of life and in accordance with contextual factors that can contribute to determine, not only which t emotional tendency is active at any one time, but also the interplay between established traits and present experience (Arciero and Bondolfi, 2009). Notice that this model does not presuppose the development of one affective disposition to the exclusion of the other. We can say that some emotions are hypercognized (Levy, 1973).

### THE INWARD DISPOSITIONAL AFFECTIVE STYLE PROPERTIES

The more those relationships generate specific episodes and interactions which the organism is biologically ready to interpret as being relevant to survival, the more the emoter’s response will involve the activation of basic emotions (Ekman and Cordaro, 2011). As a result, forms of reciprocity actualized in recurrent situations eliciting basic emotions induce the emoter to structure emotional disposition focused prevalently on basic emotions (Bertolino et al., 2005; Rubino et al., 2007). Recurrent activation of basic emotions in response to a stimulation on the part of significant others, guides the emoter’s perception of personal stability according to a frame of reference that employs a predominantly body-centered coordinate system (Arciero et al., 2004; Mazzola et al., 2010). This is the inward tendency which characterizes prevalently those people whose common distinguishing trait is the search for stability, assigning priority to the understanding of the gut aspects of emotions in their relationships with others and with the world (Arciero, 2002, 2006; Arciero and Bondolfi, 2009). Such strong interoceptive polarization corresponds to an equally strong attention toward a range of situational aspects that may lead to an alteration of the baseline interoceptive-emotional level of the subject (Wiens et al., 2000; Barrett et al., 2004). While the person’s feeling of interoceptive stability here acts as the reference system regulating his/her position with respect to the world and others, it also opens possibilities for action aimed at maintaining stability itself: for instance, the pulling or pushing away from certain people, situations or contexts, always for the sake of stability (Katkin et al., 2001; Arciero, 2002, 2006; Arciero and Bondolfi, 2009). Another equally significant attitude of this affective disposition is the need to face and anticipate conditions that may alter stability by generating fields of action to buffer environmental stimuli, e.g., the need for predictability (Arciero, 2002, 2006; Arciero and Bondolfi, 2009). Thus the subject’s dialectic with alterity here centers on a range of interoceptive modulations that both secure a stable perception of self – by bestowing meaning upon one’s strength or frailty – and allow the individual to manage and foresee contingent situations, to the point where s/he is able to avoid what he deems as excessively activating circumstances (Arciero, 2002, 2006; Arciero and Bondolfi, 2009).

### THE OUTWARD DISPOSITIONAL AFFECTIVE STYLE PROPERTIES

Unlike basic emotions, those emotions which emerge through mediated affective engagement, because of their limited viscerality, can change more quickly and more easily, since they tax the system’s visceral resources less (Lutz, 1988; Draghi-Lorenz et al., 2001). Such changeability favors the development of greater flexibility with regard to the flow of ongoing events (Gergen, 1991). This outward affective disposition mainly characterizes those people who construct a sense of personal stability through time by anchoring their identity to external reference points (persons, contexts, or abstract system of coordinates), attempting to synchronize their feelings with those points (Van Baaren et al., 2003; Ashton-James et al., 2007). Focusing on an external frame accounts for the reduced and sometimes aspecific viscerality of the emotional states perceived, bolstering the development of the cognitive dimension of emotion (Dalgleish and Power, 1999). It also explains the sense of emptiness of the emotional states perceived by the person, or the sensation of being nothing which some of these people may experience in relation to the loss of reference points which support the sense of one’s continuity over time (Arciero et al., 2004; Arciero and Bondolfi, 2009). What is most evident from this other perspective is that alterity – understood as a type of anchorage used to maintain one’s stability over time (people, contexts, images, thoughts, rules, etc.) – becomes the source of information to recognize one’s own emotional experience, hence becoming part of that experience (Chartrand and Bargh, 1999). Think, for example, of how, by way of engagement with the other, socially aware emotions come into being, including: ambivalence, ambiguity, indefiniteness, vagueness, complicity, yieldingness, complacency (Lutz, 1988). On the other hand, a sense of guilt and void arises from the subject’s lack of the reference systems (Arciero and Bondolfi, 2009).

Taking part in a discourse can also give rise to a reference system. As highlighted by Davies and Harré (1990), an individual can emerge through the processes of social interaction, not as a relatively fixed end product but as one who is constituted and reconstituted through the various discursive practices in which they participate. Any particular conversation is understood in terms of someone taking on a certain role. With positioning, the focus is on the way in which the discursive practices constitute the speakers and hearers in certain ways and yet at the same time is a resource through which speakers and hearers can negotiate new positions (Davies and Harré, 1990). In this respect, the positioning that occurs during the discourse provides the participant with a source of information to recognize one’s own emotional experience.

As we have emphasized, one perceives alterity and simultaneously co-perceives oneself. This might explain why the so-called interdependent self-construal disposition tends not only to assimilate others self cognitively, emotionally, and perceptually (Markus and Kitayama, 1991; Stapel and Koomen, 2001), but also why it tends more to non-consciously mimic others’ habitual movements than the independent self-construal disposition does (Van Baaren et al., 2003; Ashton-James et al., 2007). However, it might also account for differences between groups in emotional empathy. Indeed, in a study by Sonnby-Borgström (2002), high-empathy subjects were found to exhibit a higher degree of mimicking behavior than low-empathy subjects when exposed to pictures of angry and happy faces.

### EMPIRICAL EVIDENCE FOR INWARDNESS AND OUTWARDNESS

According to this theoretical approach, our underlying assumption in affective neuroscience research is that the two dispositional affective styles can contribute to account for interindividual variability in brain activity. That is, individual differences in structuring the affective domain can be associated to the activation of different neural areas in response to the same emotional stimuli. In our first functional magnetic resonance imaging (fMRI) study (Bertolino et al., 2005) the inward and outward participants were presented with a series of images, consisting of three fearful faces (two of which were identical), and were asked to identify which of the three faces were identical. The two groups of subjects were also comparable with regard to the genetic features of the serotonin transporter which, as demonstrated by Hariri et al. (2003), may modify the activity of specific neuroanatomic structures, such as the amygdala during the implicit processing of faces expressing fear. In line with our hypothesis, inward subjects exhibited greater activation in the amygdala and the mesial prefrontal cortex. On the other hand, outward subjects exhibited more intensive activation in the fusiform gyrus, the associative occipital cortex, and the dorsolateral prefrontal cortex. These results confirmed the initial hypothesis, that the emotional makeup triggered by the same stimuli was different in the two groups since the activity in some regions of the neural networks involved was not the same. To illustrate, inward subjects activated neural circuits primarily associated with fear in general and with its visceral correlates (amygdala), which leads one to surmise – in people with this kind of affective disposition – a more pronounced sensitivity to stimuli giving rise to alarm. Outward subjects, instead, activated areas assigned to the recognition of facial features, in addition to those allocated to the integration of emotions and cognitive functions, orienting the subject toward a greater sensitivity to cold facial features. It is noteworthy that the different amygdala reactivity in the two groups was not explained by the serotonin transporter genotype. These results suggest that aspects of dispositional affective style are rooted in biological responses of the fear circuitry associated with the processing of environmental information. A subsequent fMRI study of (Rubino et al., 2007) showed that the explicit recognition of fearful and angry faces (cognitive labeling) elicited different areas of activation in inward and outward. More specifically, inward subjects exhibited far greater engagement of the medial PFC (BA 9), whose activity is associated with cognitive aspects that are closely related with emotional processing.

In a further fMRI study (Mazzola et al., 2010), we hypothesized that such dispositional affective styles could be affected by those brain areas engaged in response to pain empathy. To address this question, we designed an experiment aiming to gage the effect produced by observing others’ pain. Inward and outward participants were exposed to highly self-related visual stimuli: images of their partner’s faces, in both painful and neutral situations, and of unknown faces, in both painful and neutral situations. Looking at a painful facial expression in one’s partner elicited greater activation in the left posterior insula/BA13 in inward, whereas in the left precuneus/BA31 and right medial prefrontal cortices/BA10 in outward. Consistently with our hypothesis inward had greater involvement of an area such as the posterior insula associated with regulating bodily states. On the contrary, the outward group demonstrated activation in precuneus (and interconnected posterior cingulate) and medial prefrontal cortices which are engaged in continuously gathering and visualizing concurrent information on the self and the external world (co-perception) as well as in the assessment of self-relevant sensations. It would thus seem evident that the different ways in which onlookers structure their feelings of personal stability is reflected in the difference in recruiting the brain circuits which are elicited when empathizing with the pain felt by one’s partner (Mazzola et al., 2010).

The aim of the present study was to improve the means at our disposal to describe inward and outward dispositions and to discriminate between people with higher inward and outward pronenesses. Accordingly, the focus will be on the prevalent properties of people’s affective engagement with oneself, the world, and others. The constructs are discussed separately and in their extreme. Of course, in individuals, what seem to be reified here exist only as tendencies of varying strength.

The objectives of the analysis may be divided in two broad classes: (1) item development, scale construction, and assessment; (2) assessment of reliability, factorial validity, and construct validity. In the following section the procedures used for item writing, data collection, and analysis are described in detail.

## MATERIALS AND METHODS

### CONSTRUCTING ITEMS FOR THE DASQ

Items were developed to measure the emotional makeup composing inward and outward dispositional affective styles, as previously described. A pool of suitable items was created by describing inward and outward attitudes toward life situations. It included items which referred, for instance, to the need for consent and approval, sensitivity to judgment, and vulnerability to criticism (e.g., “When I am not considered by partners I feel a sense of emptiness”) as well as items which referred to the need for emotional over-control in situations which, otherwise, would be felt as potentially dangerous (e.g., “I feel fragile when things are beyond my control”). The items were written clearly and concisely in order to tap dispositions in the realm of a common person’s experience. Each item was then w rated high or low by two experts in a blind fashion with respect to their relevance vis-à-vis to the constructs as we have defined. Concordance between the two experts was 100%. According to the dispositional affective styles, two scales were then established: Self-centric engagement (SCE; 25 items) and Other-centric engagement (OCE; 35 items). The 60 potential items were randomly ordered as a single scale. Respondents were asked to rate on a 7-point scale (1 = completely false, 7 = completely true) the representativeness or frequency of each item in their current time of life.

### FACTOR STRUCTURE

One of our aims was to assess the validity of inward and outward scales. We hypothesized an oblique two-factor structure to explore the data. We expected a correlation between the two-factors because of our model assumptions. Indeed, the two scales referred to the two general constructs that are not mutually exclusive but are partially overlapping. For instance, sensitivity to judgment can overlap with a need for emotional over-control.

### CONSTRUCT VALIDITY

Our second purpose was to differentiate participants with higher or lower proneness on each of the two constructs – inwardness and outwardness. Consistent with our assumptions, we predicted a slight correlation between the two scales, and that the high inward respondents would score higher on the SCE scale, whereas the high outward respondents would score higher on the OCE scale. This question was addressed by assessing the predictive performances of the scales. Further details are given in the following sections.

In addition to examining construct validity, we also investigated the correlations between the In-Out dispositional affective style questionnaire (IN-OUT DASQ) and personality traits using the Big Five Questionnaire (BFQ; Caprara et al., 1993), and affective tendencies using the Positive and Negative Affect Schedule (PANAS; Watson et al., 1988).

### SEMI-STRUCTURED INTERVIEWS

In order to assess the construct validity, the participants were classified in low vs. high inward, and low vs. high outward through a semi-structured interview which was administered independently by two trained investigators who were blind to each other’s results. The aim of the semi-structured interview was to reconstruct the sense of personal stability by the assessment of emotional activation, duration and regulation in two meaningful emotional experiences triggering anger and fear. The steps of the semi-structured interview have been described in detail in our previous study (Mazzola et al., 2010). In order to classify participants, the duration criterion was employed to define high vs. low inwardness, as well as high vs. low outwardness. The longer the duration of triggered emotions, the higher the level of inward proneness. The more rapid the change toward a different point of reference, the higher the level of outward proneness.

### SAMPLE AND PROCEDURE

An initial exploratory factor analysis (EFA) was conducted in a random sample of 292 Italian volunteers with various types of job, possessing different educational qualifications and being of various ages (135 females; median of age = 32 years IQR = 14), balanced by level of education (46% with 18 or more years of education, 55% with 13 years of education). Volunteers were recruited through flyers at places of work and universities, and through word of mouth by colleagues and collaborators of our Institute. Exclusion criteria included any significant psychiatric conditions as evaluated with the Structured Clinical Interview (SCID). Participants were asked to respond to the items on the basis of the following instructions: “There are no right or wrong answers for the following questions. Please answer based on your most frequent way of being, feeling, and behaving.” In order to avoid any potential effect of interactions between the level of anxiety and the inability to identify and describe personal affective states, after completing the IN-OUT DASQ participants also responded to the State-Trait Anxiety Inventory (STAI; Spielberger, 1983) and to Toronto Alexithymia Scale (TAS-20; Bressi et al., 1996; Taylor, 2000). Once the participant had completed the questionnaires, the semi-structured interview was administered by the first trained investigator (VM). In order to assess the invariance in scores over time, the participants filled out the retest 2 months later. Following this, the semi-structured interview was administered by the second trained investigator (GA) who was blind to his colleagues’ results. Only two participants did not return the retest within the deadline. The number of questionnaires returned with at least one missing response was 30 (10.3%) in the test and 5 (1.7%) in the retest. In the test questionnaire, responses to 45 items (out of 60) were missing, with a maximum frequency of 4 for a single item. Therefore, no specific missing data pattern was evident. However, since it was not possible to make a realistic assumption about the non-response mechanism, incomplete questionnaires were not included in data analysis. The final sample consisted of 256 participants who filled out the questionnaires completely. The factor structure of the final scales (14 items) was assessed in a second sample of 300 participants (180 females; median of age = 24 years IQR = 17, 33% with 18 or more years of education, 48% with 15 years of education, 19% with 13 years of education) through confirmatory factor analysis (CFA) techniques. Participants filled out the IN-OUT DASQ, STAI, and TAS questionnaires. The assessment of correlations of SCE and OCE scales with BFQ and PANAS was performed on a third sample of 153 participants (102 females; median of age = 33 years IQR = 10).

The study was approved by the Comitato Etico Indipendente Locale of the Azienda Ospedaliera “Ospedale Policlinico Consorziale” of Bari. Informed written consent was obtained from all participants before participation.

### DATA ANALYSIS

#### Scale construction

In a preliminary step of the analysis, frequency distributions were examined for each item to identify problematic patterns, such as extreme skewness, mean scores near the top or bottom end of the Likert scale and limited variability. In order to identify excessively redundant item pairs, inter-item correlations were also examined. Then, the remaining items were used for the development of the SCE and OCE scales. The unidimensionality of each scale was ascertained through an item-level EFA. The items were selected through a forward step procedure, in which at each step the item that determined the maximum increase of Cronbach’s alpha was included in the scale. Scores for each scale were obtained by summing responses to all items.

#### Scale assessment

The factorial structure of the final scales was assessed through an item-level EFA, using the principal axis extraction method. Two analyses were conducted to assess separately the internal consistency of each scale. In addition, an analysis was carried out on the entire item pool to assess the overall factor structure. The number of factors was determined by examining the scree plot and retaining the factors corresponding to the first larger eigenvalues, until the slope of the graph changes from rapid to slow decline. In addition, a more formal approach was employed by assessing the minimum average partial (MAP) criterion (Velicer, 1976). In the overall analysis, oblique axes were determined by the Promax rotation method. For each scale, internal consistency reliability was assessed through Cronbach’s alpha. Test–retest reliability was assessed through the Intraclass Correlation Coefficient (ICC).

Construct validity was assessed by evaluating the performances of the scale scores in differentiating the groups of participants ascertained by the semi-structured interviews, namely high inward vs. low inward, and high outward vs. low outward. To this aim, we used the area under a receiver operating characteristic (AUROC) curve (Swets, 1988). The AUROC is an assessment of the discriminatory power of the distributions of the scores within the groups, with 1.0 being perfect discrimination between groups and 0.5 being random discrimination.

In order to evaluate the predictive performances of the scales, the classification of the participants into two mutually exclusive groups were determined by dichotomizing scale scores, and compared with the groups delineated as a result of the semi-structured interviews (DeVellis, 2011). Several classifications were obtained by using distinct cut-off values to dichotomize the scores. The selection of the “best” one was based on the evaluation of misclassification rates and of the agreement between dichotomized scores and reference classifications as determined by semi-interviews. The proportions of misclassified subjects were evaluated for each classification. For instance, for the division between low inward and high inward, the misclassified subjects were high inward classified as low inward, and low inward classified as high inward. The agreement was evaluated using Cohen’s Kappa index.

The initial factor structure identified in the EFA provided the hypothesized factor structure for the separate CFA performed on a second sample. WLSMV (weighted least squares means and variance adjusted) estimation method was used to account for non-normality of item distributions. The model fit of three factor solutions: (1) one factor; (2) two orthogonal factors for SCE and OCE items respectively; (3) two oblique factors; was compared by root mean square error of approximation (RMSEA), Bentler’s comparative fit index (CFI), and Tucker–Lewis index (TLI). Acceptable fit of the model was defined as: RMSEA < 0.1, and CFI, TLI > 0.95.

The association of SCE and OCE with BFQ and PANAS scales was assessed through bivariate correlations and semipartial correlations (Cohen et al., 2013). Semipartial correlations were calculated by removing the shared variance of SCE and OCE, thus allowing to evaluate the association of unique parts of inward and outward dispositions with the constructs measured by BFQ and PANAS scales.

All analyses were carried out using the R software (R Development Core Team, 2013) with the psych package (Revelle, 2013).

## RESULTS

### ITEM SELECTION

The initial pool consisted of 60 items divided into the SCE scale (25 items) and the OCE scale (35 items). The average SCE scores ranged from 2.50 to 5.33 and standard deviations from 1.37 to 1.97. The degree of skewness was overall admissible, with values from -1.16 to 0.99. Cronbach’s alpha was equal to 0.897, close to the value of McDonald’s Omega, 0.903 (McDonald, 1978). The ICC was 0.915 [95% confidence interval (CI): 0.892, 0.933]. On the other hand, the average OCE scores ranged from 2.47 to 5.42, and standard deviations from 1.38 to 1.92. The skewness ranged from -1.13 to 0.91. Cronbach alpha, McDonald omega, and ICC were 0.931, 0.933, and 0.917 (95% CI: 0.895, 0.935) respectively.

After the preliminary verification of item distributions, the item pool included 39 items, 14 of the SCE scale, 25 of the OCE scale. The excluded items had very weak (13 items) or not expected (seven items) values of inter-item correlations. The only redundant pair of items (with a correlation higher than 0.8) was excluded to avoid an inflated estimated reliability. In the selected pool of SCE items, inter-item correlations ranged between -0.117 and 0.599, with an average value of 0.291, whereas in OCE pool the range was between -0.174 and 0.732, with an average value of 0.282.

The two separate EFAs yielded 11 SCE items and 16 OCE items with loadings greater than 0.4 in absolute value on the first factor, thus showing an appreciable internal consistency of the two scales. The final scales included seven items each (**Tables 1** and **2**). All the items included in the final SCE scale described the need for emotional over-control in situations that, otherwise, would be felt as potentially dangerous. On the other hand, the final OCE items referred to the need for approval, and sensitivity to judgment.

**Table 1:** Assessment of number of factors.

Component number	SCE		OCE		SCE + OCE	
	Eigen-values	MAP criterion	Eigen-values	MAP criterion	Eigen-values	MAP criterion
1	3.77	0.036	3.92	0.040	6.49	0.022
2	0.77	0.076	0.74	0.094	1.29	0.021
3	0.60	0.138	0.67	0.153	0.87	0.031
4	0.59	0.262	0.55	0.253	0.74	0.042
5	0.49	0.439	0.51	0.490	0.68	0.056
6	0.44	1.000	0.35	1.000	0.61	0.075
7	0.35	–	0.26	–	0.59	0.100

*Reported in the table are the eigenvalues and averages of squared partial correlations (MAP criterion) for the SCE and OCE scales and for the whole item pool (SCE + OCE).*

**Table 2:** Item factor analysis for the SCE and OCE scales.

	Test					Retest				
	Separated FAs		Overall item pool			Separated FAs		Overall item pool		
SCE scale	PA 1	h^2^	PA 1	PA 2	h^2^	PA 1	h^2^	PA 1	PA 2	h^2^
(1) I feel fragile when things are beyond my control.	0.77	0.60	0.33	0.44	0.61	0.75	0.57	0.34	0.43	0.58
(2) Sometimes I imagine dangerous situations that threaten the safety of myself or my loved ones.	0.65	0.43	0.16	0.44	0.43	0.62	0.38	0.16	0.42	0.38
(3) Realizing I have constraints makes me feel “stuck.”	0.66	0.44	0.21	0.41	0.43	0.65	0.42	0.20	0.42	0.41
(4) I must always be sure that if I were to get sick there would be someone to help me.	0.66	0.43	0.15	0.45	0.45	0.63	0.40	0.14	0.45	0.42
(5) I always need to know where the people dearest to me are.	0.58	0.34	0.16	0.39	0.36	0.60	0.36	0.14	0.43	0.40
(6) The unexpected breakup of a significant relationship makes me feel in danger.	0.68	0.47	0.29	0.39	0.47	0.64	0.41	0.32	0.35	0.44
(7) When I find myself trapped in a situation, I have intense reactions of anger.	0.74	0.54	0.21	0.48	0.54	0.72	0.52	0.22	0.47	0.51

	**Perc Var^**a**^**					**Perc Var^**a**^**				

	46.3%					43.7%				

**OCE scale**	**PA 1**	**h^2^**				**PA 1**	**h^2^**			

(1) When I feel I am important for someone else I’m afraid to disappoint him/her.	0.71	0.50	0.47	0.19	0.51	0.64	0.41	0.43	0.18	0.41
(2) Others’ approval gives me the measure of how much I am worth.	0.61	0.37	0.42	0.13	0.39	0.66	0.43	0.47	0.11	0.45
(3) In a serious relationship I risk losing sight of what matters to me.	0.66	0.44	0.40	0.24	0.43	0.60	0.36	0.37	0.20	0.34
(4) Feeling ignored by a partner gives me a feeling of loss.	0.81	0.65	0.50	0.28	0.65	0.82	0.67	0.53	0.27	0.68
(5) If one of my actions unintentionally hurts someone, I condemn myself for not having foreseen that this might happen.	0.51	0.26	0.32	0.17	0.26	0.50	0.25	0.32	0.17	0.25
(6) When I am not considered by partners I feel a sense of emptiness.	0.80	0.64	0.51	0.22	0.62	0.84	0.70	0.55	0.22	0.68
(7) Unless I receive reassurance I feel unsure of my abilities.	0.76	0.58	0.49	0.24	0.60	0.75	0.57	0.50	0.23	0.59

	**Perc Var^**a**^**		**Perc Var^**a**^**		**PA Cor^**b**^**	**Perc Var^**a**^**		**Perc Var^**a**^**		**PA Cor^**b**^**

	49.2%		23.0%	20.7%	0.71%	48.4%		25.4%	21.4%	0.69%

*The items were translated into English only for the purpose of publication.*

*For the separated factor analyses, columns show: factor loadings on the first principal axis (PA1) and item communalities (h^*2*^); for the factor analysis of the overall item pool: reference vector correlations for the two principal axes (PA1, PA2) and item communalities (h^*2*^). ^a^Percentage of variance explained by principal axes; ^b^Correlation between principal axes.*

### FACTOR STRUCTURE

The results of the EFA analyses of the final scales are reported in **Tables 1** and **2**. For the separated analyses of SCE and OCE scales, the scree plot and MAP criterion showed the unidimensionality of each scale (**Table 1**).

In the overall analysis, a change in slope (“elbow”) was found at the third eigenvalue, thus suggesting a two-factor solution. The minimum of average squared partial correlations was reached at the second eigenvalue, thus supporting the result above. To assess the association of the items with the two oblique factors, we reported the reference vector correlations (Gorsuch, 1983), which measure the correlation of a variable with the unique part of a factor which is not predictable by other factors.

As shown in **Table 1**, the SCE items were more highly correlated with the second principal axis, whereas the OCE items were more highly correlated with the first principal axis. However, the cross correlations between SCE items and the first axis and between OCE items and the second axis were not negligible. This result revealed a moderate correlation between SCE and OCE scales, between the need of control (item 1 SCE scale), the need of others’ approval (item 2 OCE scale; *r* = 0.40, 95% CI: 0.29, 0.50, *p* < 0.0001), and the sense of guilt (item 6 OCE scale; *r* = 0.57, 95% CI: 0.49, 0.65, *p* < 0.0001). Nevertheless, there were item correlations within the two scales consistent with the core of the two dispositions investigated here. Indeed, within the SCE scale there was a higher correlation coefficient between the need of control (item 1; *r* = 0.59, 95% CI: 0.50, 0.67, *p* < 0.0001), the danger of an unexpected event (item 6), and the anticipation of dangers (item 2; *r* = 0.51, 95% CI: 0.41, 0.60, *p* < 0.0001). On the other hand, within the OCE scale is worth noting the highest correlation coefficient between the sense of guilt (item 5), the sense of emptiness (item 6; *r* = 0.36, 95% CI: 0.24, 0.46, *p* < 0.0001), and the need of approval (items 1 and 2; *r* = 0.42, 95% CI: 0.31, 0.51, *p* < 0.0001; *r* = 0.32, 95% CI: 0.21, 0.43, *p* < 0.0001 respectively).

In the test sample, the correlation between the two principal axes was equal to 0.71, slightly higher than expected. However, such a correlation between SCE and OCE items may have been overestimated because of common method bias, i.e., an inflation of relationships by shared method variance (Conway and Lance, 2010). This consideration led us to conduct a further examination of the factorial dimensions measured by the overall item pool, in which common variance was accounted for through the use of a “*post hoc*” technique (Podsakoff and Organ, 1986; Podsakoff et al., 2003). A principal component analysis without rotation was performed on the 39 items which were used to develop the scales. According to the assumption that the first factorial axis included the best approximation of common variance, the goal was the assessment of latent constructs through the examination of the successive axes.

**FIGURE 1:**
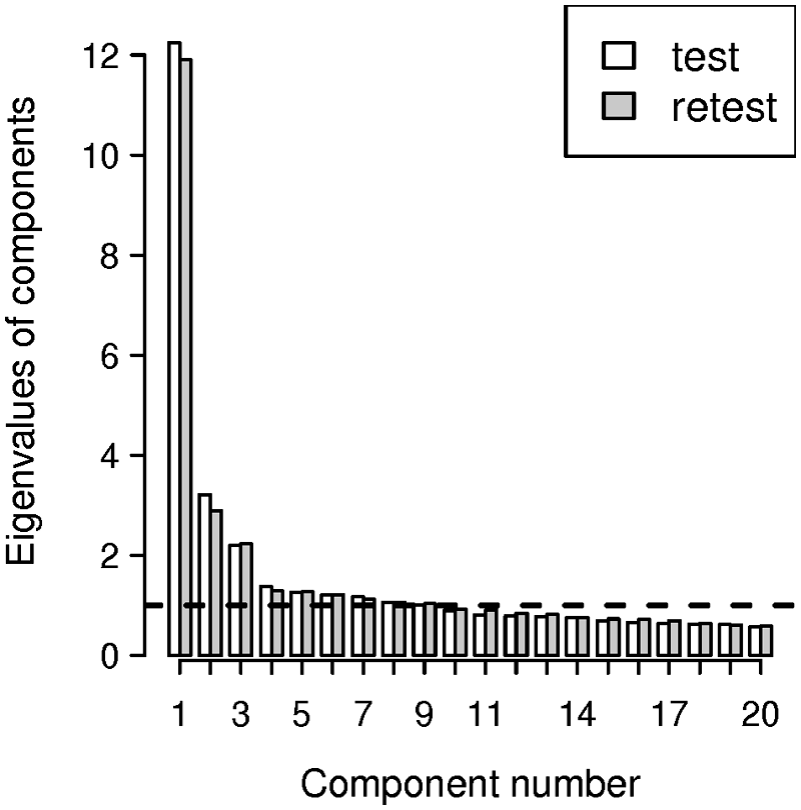
**The scree plot of the exploratory factor analysis of the In-Out Dispositional Affective Style Questionnaire (IN-OUT DASQ) item pool.** The number of factors is determined by examining until the slope of the graph changes from rapid to slow decline. The scree plot suggests a three factor solution: one “method” factor and two “latent trait” factors.

**FIGURE 2:**
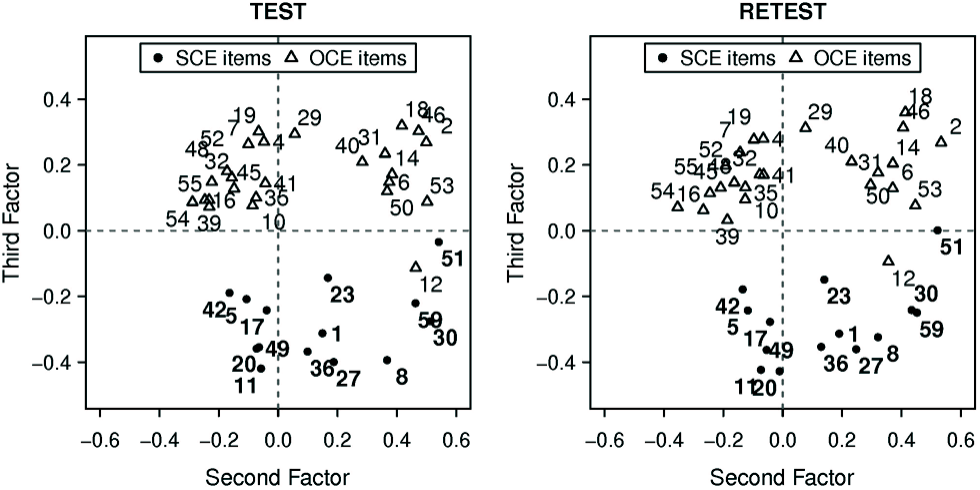
**Item loadings on the second and third factorial axes.** The points represent the selected Self-centric engagement (SCE) items and Other-centric engagement (OCE) items respectively, forming two separated clusters. The proximity of the points of each cluster reveals a pattern of correlation of the projections of items on the plane.

Consistent with the hypothesized existence of one “method” factor and two “latent trait” factors, the scree plot suggested a three factor solution (**Figure 1**). The factor loadings on the second and third factorial axes are shown in **Figure 2**. The points representing the selected SCE items and OCE items respectively formed two quite separate clusters. The proximity of the elements of each cluster revealed a pattern of correlation of the projections of items on the plane spanned by the second and third axes, thus providing some evidence of internal consistency for both SCE and OCE items. We did not investigate further results (factor variances, item correlations) because *post hoc* procedures are not suitable for assessing them (Kemery and Dunlap, 1986; Podsakoff et al., 2003; Conway and Lance, 2010). In conclusion, we found evidence of two latent factors after having accounted for common variance. The results here discussed were in agreement with those previously illustrated concerning the overall factorial structure of SCE and OCE scales.

### RELIABILITIES, DESCRIPTIVE STATISTICS, AND SCALE CORRELATIONS

The descriptive statistics and reliability coefficients are reported in **Table 3**. The values of Cronbach’s alpha, McDonald’s omega, and ICC provided robust evidence of reliability for both the two scales. The correlations between SCE and OCE were moderately high in magnitude (test data: *r* = 0.68, 95% CI: 0.61, 0.75, *p* < 0.0001; retest data: *r* = 0.66, 95% CI: 0.59, 0.73, *p* < 0.0001) and close to the correlations between principal axes obtained in previous analyses.

**Table 3:** Reliability.

SCALE	Mean	SD	Alpha	Omega	ICC
**SCE (seven items)**
Test	25.4	9.1	0.855	0.857	0.929 (0.910, 0.944)
Retest	25.7	9.5	0.867	0.844	
**OCE (seven items)**
Test	27.5	8.4	0.843	0.869	0.913 (0.889, 0.931)
Retest	28.0	8.6	0.862	0.865	

*Statistics reported for each scale: mean and standard deviation (SD), Cronbach’s alpha, McDonald’s Omega, Intraclass Correlation Coefficient (ICC) for assessing test–retest reliability.*

### CONSTRUCT VALIDITY

According to the semi-structured interviews, the 256 participants consisted in 62 high inward and 194 low inward, and 99 high outward and 157 low outward. The sample proportions of high inward and high outward were 24.2% (95% CI: 19.4, 29.8%) and 38.7% (95% CI: 32.9, 44.8%) respectively. We expected a priori that high inward would have scored higher on the SCE scale, and high outward would have scored higher on the OCE scale.

**FIGURE 3:**
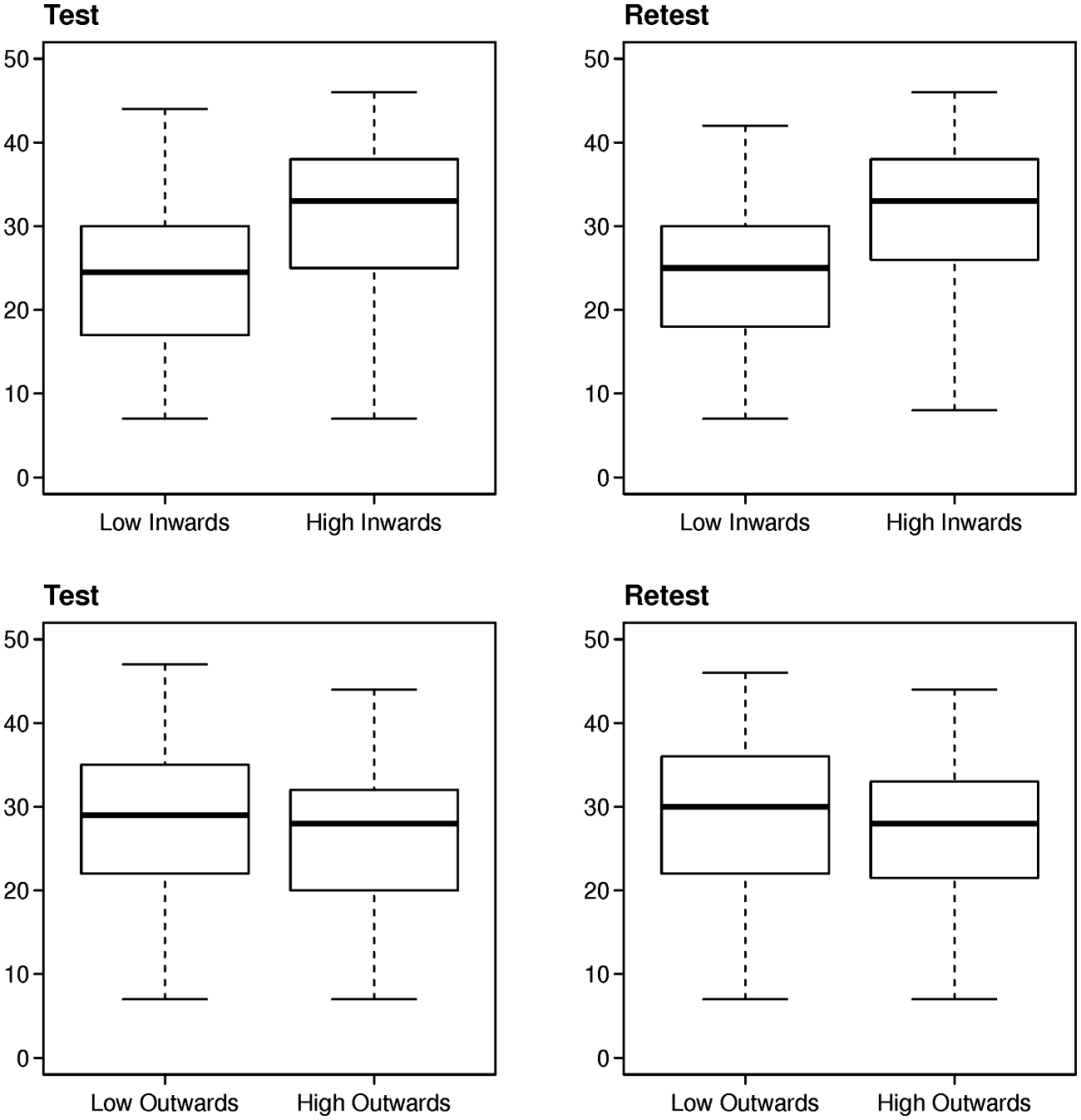
**Distributions of Self-centric engagement (SCE) and Other-centric engagement (OCE) scores.** The box plots show that SCE scores are more separated between high and low inward than OCE scores.

Therefore, the distributions of scale scores in the target groups were evaluated. The box plots showed that SCE scores were rather separated between high and low inward, whereas OCE scores did not differentiate between high and low outward (**Figure 3**). The average scores of the SCE scale were 31.3 [standard error (SE): 0.56] and 23.5 (SE: 0.52) in high and low inward respectively. The difference in SCE scores between the two groups was significantly greater than zero (Mann–Whitney test: *p* < 0.0001). About the OCE scale, the average scores were 26.2 (SE: 0.58) and 28.2 (SE: 0.59) in high and low outward respectively. The difference between the two groups was significant in the test data (*p* = 0.01) but not in the retest (*p* = 0.3). Thus, there was poor evidence supporting the second expectation. Accordingly, further analyses were not carried out for the OCE scale.

In the examination of the AUROC the predictive performances of the scales were deemed significant when the respective 95% CIs did not include the value of 0.5, which is made by chance. Results are shown in **Table 4**. The SCE scale showed significant moderately high performances in differentiating inward in agreement with our first expectation.

**Table 4:** Assessment of construct validity.

Scales	High-inward vs. low-inward		
	Area under ROC curve	Proportion of misclassifications	
		Low-inward (%)	High-inward (%)
SCE (test)	0.739 (0.685, 0.794)	5.7 (3.0, 9.5)	61.3 (48.9, 72.8)
SCE (retest)	0.736 (0.677, 0.794)	8.8 (5.3, 13.3)	58.1 (45.6, 69.8)

	**High-outward vs. low-outward**		
	**Area under ROC curve**	**Proportion of misclassifications**	

		**Low-outward (%)**	**High-outward (%)**

SCE (test)	0.634 (0.590, 0.679)	14.0 (9.2, 20.0)	66.7 (57.0, 75.4)
SCE (retest)	0.612 (0.567, 0.657)	24.2 (18.0, 20.0)	55.6 (45.7, 65.1)

*Reported in the table are: the area under the ROC curve with 95% confidence intervals, and the proportions of misclassified participants, with 95% confidence intervals.*

Regarding the classification of participants based on scale scores, a nearly moderate agreement (κ = 0.39) was observed between the semi-structured interviews and the OCE scale classification, although with a rather high error in classifying high inward. Further investigations are required to refine the assessment of this trend.

Next, the bivariate correlations between the DASQ, BFQ, and PANAS scales were examined (**Table 5**). The SCE scores showed a moderate negative correlation with Neuroticism (*r* = -0.62, *p* < 0.01) and a moderate positive correlation with Negative Affect (*r* = 0.48, *p* < 0.01), though the semipartial correlations were weaker (*r* = -0.45, 0.27, *p* < 0.01). On the other hand, OCE scores showed a weak to moderate negative correlation with Neuroticism (*r* = -0.42, *p* < 0.01) and a positive correlation with Negative Affect (*r* = 0.42, *p* < 0.01), even if the semipartial correlations were both not significant (*r* = -0.05, 0.15, *p* > 0.05).

**Table 5:** Bivariate correlations and semipartial correlations of SCE, OCE with BFQ and PANAS scales.

Scales	SCE	OCE
**BFQ**
Extraversion (E)	-0.06 (0.05)	-0.16* (-0.16)
Agreeableness (A)	-0.20* (-0.19*)	-0.08 (0.05)
Consciousness (C)	-0.08 (-0.09)	-0.01 (0.05)
Neuroticism (S)	-0.62** (-0.45**)	-0.42** (-0.05)
Openness (M)	-0.09 (0.00)	-0.14 (-0.11)
**PANAS**
Positive affect	-0.03 (0.07)	-0.14 (-0.16)
Negative affect	0.48** (0.27**)	0.42** (0.15)

Semipartial correlations are reported in parenthesis. *p < 0.05, **p < 0.01.

### CONFIRMATORY FACTOR ANALYSIS

The CFA was performed using the final SCE and OCE items. The oblique two-factor solution showed the best fit (RMSEA = 0.062, CFI = 0,921, TLI = 0.906) compared to the others (one-factor: RMSEA = 0.077, CFI = 0,878, TLI = 0.856; two orthogonal factors: RMSEA = 0.166, CFI = 0,433, TLI = 0.330). These results showed a moderate fit with the hypothesized oblique two-factor structure model.

## DISCUSSION

The aim of the present study was to construct a research tool to describe inward and outward dispositions and to individuate the highest level of proneness between the inward and outward dispositional affective styles. Two scales of seven items each were finally assessed: the SCE and the OCE, respectively as a disposition index for inwardness and outwardness. Our predictions were partially confirmed, with some unexpected results.

According to our theoretical assumptions, inward and outward dispositions were operationalized as unipolar constructs. Consistent with this contention, the EFA confirmed a two-factor solution providing evidence for the unidimensionality of the SCE and OCE scales. This result was moderately confirmed by the CFA. Both the factorial analyses revealed a high correlation between these two scales. As expected, this correlation in itself is not in contrast with our theoretical assumptions. Indeed, these two general dispositions should not be thought as categorical stances that people take in an exclusive way. As general tendencies, these engagements with himself/herself and with others can be combined from a minimum to a maximum degree, in different nuances, and differently over time. Indeed, the need of control, the need of others’ approval, and the sense of guilt are all attitudes that can coexist. We controlled also for a potential confounding latent factor coming from a common variance which may account for such a correlation. Although “*post hoc*” techniques may be deficient, the partial correlation analysis confirmed that the two scales have their own internal consistency.

The results concerning the discriminant capacity were encouraging. Although our current aim was ambitious, these findings showed at least a trend in the expected direction. As we predicted, the SCE scale discriminated against high inwardness. Indeed, high inward respondents scored higher than low inward respondents in SCE. The correlations between the SCE items were fully consistent with the inwardness construct. Indeed, there was evidence of a higher correlation between the sense of frailty, danger and its anticipation, and the need of control of loved ones. The meaning of these correlations refers to the core traits of this predominant modality of emotional engagement with the self and with the environment. On one hand, it showed that the more respondents were inwardly prone, the more emotional disposition was prevalently focused on a state of alarm from which fear can be suddenly elicited. It revealed the need to face and anticipate conditions that may alter a personal stability by generating the anticipation of potential dangers and increasing a sense of control. On the other hand, the high outward respondents scored lower than low outward respondents on the SCE. According to our theoretical assumptions, this result provides evidence about the inverse relationship between high inwardness and high outwardness.

Unfortunately, our expectations concerning the OCE scale were only partially confirmed. The correlations between the OCE items were consistent with the outwardness construct. Indeed, there was the highest correlation between the feeling of guilt, the sense of emptiness, and the feeling of personal loss, as well as between the need of approval. All these correlations assessed central themes of the OCE disposition. The “guilt-emptiness-feeling of personal loss” correlation is certainly one of the most significant for understanding outwardness. Because these subjects keep their the sense of personal stability by anchoring their identity to others as external reference points, the possibility of losing this reference system can give way to a feeling of emptiness. As is known, the loss of a significant person gives way to a feeling of guilt and of emptiness. People more prone to the outwardness experience more frequently have feelings of guilt and void when they lose the other as a source of reference. This can generate a wide spectrum of discomfort, even a depressive disorder. On the other hand, the correlation between the need of approval and getting lost in a partner refers to the attempt to synchronize their feelings with others as external reference points.

Nevertheless, the OCE discriminant capacity was poor as showed by the ROC curve. Although the OCE scale revealed some basic attitudes at the core of outwardness, it was not sufficiently sensitive to individuate those that better fit to high levels of outwardness. This result might refer to the interaction between the wording of the items and the outward subject’s sensitivity to discourse as a source of self-reference. We speculate that it is as if the respondents had felt “positioned” by some items. Indeed, outward respondents, both high and low, tended to give a neutral score to those items which were worded in a more direct way.

The robust reliability suggests a good temporal stability of both the measures and the phenomena. It helps to increase the robustness of the scales and their development. Due to the multifaceted structures of the inwardness and outwardness, the sensitivity of the measures employed can be improved also by using those means that estimate other dispositions in other domains such as cognitive, motivational and interpersonal attitudes. Indeed, though the scores on the DASQ were found to be distinct from BQF and PANAS constructs, further research is required to assess other relationships in order to place OCE and SCE scales within a broader nomological network.

We acknowledge that the present study has some limitations. First, the low discriminant capacity of the OCE scale means that it cannot be used to estimate high vs. low outwardness without the semi-structured interview. We argue that the presence of neutral response undermined the OCE items more than SCE items. Since it was an exploratory study, we made this choice to avoid forcing a response. On one hand, this choice allowed us to better explore general dispositions in a large sample. On the other hand, it might have interacted with the outwardness subject’s sensitivity. In this respect, the higher outward respondents might have a more marked attitude to use the questions as a temporary source of information for recognizing their own emotional experience. This could have influenced the discriminative capacity of the OCE scale. It could be that this phenomenon might be better controlled through items worded both positively and negatively within the OCE scale. The SCE scale did not assign a priority to the understanding of the gut aspects of emotions in relationships with others and with the world. Since it is a more marked sensitivity in inwardness, this may constitute a limitation. Although such interoceptive awareness requires the investigation of behavioral tasks (e.g., hearth beat detection task), the SCE scale could be improved by the enhancement of the interoceptive sensitivity.

Overall, our previous findings indicate that differentiating the high level of inward and outward tendency in relationships with others and the world is a potential strategy for understanding and measuring individual differences. In the present study, we explored a means which is useful in this field of empirical research. It is important to bear in mind, however, that though our results provide evidence for the two constructs and their reliability, further studies are required to confirm the factorial model and its validity, also taking into account their multifaceted structures. In addition, the relationships between personality traits and dispositional affective styles still require investigation. Since this subject is many-sided, we started at the most basic level, i.e., testing a first model. Therefore, it would be suitable to continue the research line at a more complex level by including new hypotheses and new models. The questionnaire still required a proper psychological assessment (interview). Far from being exhaustive, the present study is an attempt to address the issue of individual differences from an affective dispositional point of view.

## Conflict of Interest Statement

The authors declare that the research was conducted in the absence of any commercial or financial relationships that could be construed as a potential conflict of interest.
